# A Bifactor Model of Childhood Adversity in a Longitudinal South African Birth Cohort Study

**DOI:** 10.1177/10731911251340221

**Published:** 2025-05-29

**Authors:** Joannes S. H. de Leeuw, Marilyn Lake, Milton Gering, Nadia Hoffman, Kirsten A. Donald, Heather J. Zar, Dan J. Stein, Susan Malcolm-Smith

**Affiliations:** 1 University of Cape Town, South Africa; 2 University of Witwatersrand, Johannesburg, South Africa

**Keywords:** Bifactor model, adversity, childhood, community violence, distress response, LMIC

## Abstract

Early exposure to violence can elicit a toxic-stress response in children. However, not all exposures to violence exert the same negative impact. This study aimed to develop a bifactor model of childhood adversity by integrating two established measures, the Child Exposure to Community Violence questionnaire and the Pediatric Emotional Distress Scale. The Adversity Exposure-Response Model was created using caregiver-proxy report data from children aged 3.5 and 4.5 years (*N* = 801) in a South African birth cohort from two high-risk, low-income communities. A bifactor model best fit the data, with the newly formed composite serving as a statistically significant predictor of exposure to traumatic events (β = .34, *p* < .001). As predicted, this bifactor model provided a holistic approach to childhood adversity, challenging the assumption that all adverse events result in uniformly negative outcomes. It offers a comprehensive screening tool to identify at-risk children early, facilitating targeted interventions in high-risk settings.

## Introduction

### Measuring Childhood Exposure to Adversity

Adverse childhood experiences (ACEs) refer to potentially traumatic events that occur before the age of 18, such as abuse, neglect, or exposure to violence, which require significant adaptation by a child (Lacey & Minnis, 2020; McLaughlin, 2016). Early exposure to adversity can elicit a toxic-stress response in children; hence, there is a need to detect such exposure and screen for its impact to prevent future negative responses and promote positive development (Oh et al., 2018). Toxic stress, when prolonged or intense, can negatively impact children’s mental health and development, increasing the risk of long-term physical, emotional, and behavioral problems (Lewis et al., 2019). However, not all exposures to adverse events exert the same negative impact on mental health outcomes (Ellis et al., 2005). There are interindividual differences, and child development can be impacted differently by deprivation or threat (McLaughlin et al., 2014) or, for instance, by physical or sexual abuse (Ellis et al., 2005).

Operationalization of childhood adversity is predominantly done by either summing categories of adversity or by obtaining a total score that represents the frequency, severity, and/or proximity of childhood adversities (Burgermeister, 2007; Cook et al., 2022), and seems less focused on the complexities of individuals’ toxic-stress response (Lewis et al., 2019). ACEs such as childhood maltreatment (sexual, physical, or emotional abuse) and exposure to violence, are consistently associated with a twofold increase in the risk of common mental disorders and suicidality later in life (Sahle et al., 2021). Assessing childhood adversity is therefore crucial in understanding the impact on child health, since it should distinguish between adverse events that happen to a child and their stress response to these events (Boyce, 2016; Nelson et al., 2020) to develop strategies to break the intergenerational cycle of adversity and improve mental health outcomes worldwide (Sahle et al., 2021).

### Moving Beyond the Traditional Adverse Childhood Experience Framework

The ACE framework has been instrumental in advancing our understanding of how childhood adversity impacts health and development (McLaughlin et al., 2014). However, the framework has significant limitations that constrain its utility for both research and practice (Lacey & Minnis, 2020). First, the summative approach to ACE scoring assumes that all adversities exert equal effects on outcomes, overlooking the unique contributions of specific adversities and the interactions between them (Lanier et al., 2018; McLaughlin et al., 2014), making it difficult to study the mechanisms through which adversities exert their effects, (Lanier et al., 2018). Second, traditional ACE frameworks often disregard the timing, chronicity, and discontinuity of adversities, treating them as static rather than dynamic processes (Howe et al., 2015; Slopen et al., 2014), making it difficult to investigate how adversities affect children differently at various developmental stages or across cultural and socioeconomic contexts (McLaughlin, 2016; McLaughlin et al., 2014). To address these gaps, we propose a novel conceptualization of childhood adversity that moves beyond summative approaches, the Adversity Exposure-Response Model (AERM). This framework incorporates both shared and specific effects of adversities, accounts for individual differences in stress responses, and considers the broader developmental context.

### Habituation and Desensitization in Risk-Exposed Children

Children growing up in under-resourced communities in low- and middle-income countries (LMICs) are often exposed to a greater variety and multitude of risks, compared to children in high-income countries (HICs; Brittain et al., 2015; Stein et al., 2015). This highlights the need for specificity in identifying childhood exposure to adversity and especially to check for the exposures’ subsequent impact (Lewis et al., 2019). Theories such as desensitization and habituation (Rankin et al., 2009) provide valuable frameworks for understanding the (lack of) emotional response in children. For instance, when community violence is pervasive, desensitization theory posits that repeated exposure to community violence may diminish children’s emotional and physiological responses to such events over time, potentially reducing the immediate negative impact of violence on self-regulation or other developmental outcomes (Draper et al., 2023). Similarly, habituation theory suggests that through repeated exposure, children may adapt behaviorally, becoming less reactive to violence in their environments (Rankin et al., 2009). These theoretical perspectives underscore the need for a more nuanced understanding of how exposure to adversity interacts with developmental processes, going beyond simply tracking the frequency of adversity to explore its cumulative and contextual effects (Oh et al., 2018). While desensitization and habituation to violence have been well-documented in HICs, particularly concerning media exposure (Huesmann & Kirwil, 2007), there is a paucity of research exploring these phenomena in LMICs where real-life violence is rife (Ward et al., 2018). Understanding how children emotionally react and adapt to violence is, therefore, essential, given the potential long-term consequences of diminished emotional response such as lower social competence and emotion regulation difficulties (Bender et al., 2022).

### A Comparison of Models to Foreground Hidden Adversity Dimensions

Social science researchers often work with multifaceted constructs that are measured with several subscales, each intended to measure a specific aspect of that main construct, and interpretation happens through either creating a total score or looking at the subscale scores separately (Chen et al., 2012). A higher-order structure effectively captures how subdomains relate to a broader construct, but since items do not load directly onto a general factor, it limits the ability to assess both shared and unique contributions of each domain (Chen et al., 2012). Research into ACEs showed that using a broad total score complicates differentiation between type, frequency, timing, and/or impact of adversity (Hamai & Felitti, 2022) and more advanced techniques were suggested to extricate the effects of these different aspects of ACEs on childhood well-being and any negative outcomes (Cooper & Nickodem, 2021; Decrop et al., 2024). One such technique is the bifactor model, often used in intelligence research (e.g., Gustafsson & Balke, 1993; Luo et al., 1994) as well as in studies of internalizing and externalizing psychopathology (Markon, 2019) and personality disorder (Chen et al., 2012). A bifactor model suggests that in a multifaceted construct (a) there is a variability that is linked to a general factor, capturing what is common to all indicators, and (b) unique variance attributable to individual facets, representing their distinct contributions beyond the general factor (Chen et al., 2012; Markon, 2019). Recent research by Cooper and Nickodem (2021) demonstrated the utility of a bifactor model in studying childhood adversity. By incorporating multiple indicators of lifetime adversity into a multidimensional framework, their findings revealed two key insights: A general factor of adversity (e.g., overall childhood adversity) had strong predictive utility, supporting the use of a single composite score in some contexts. Further, specific factors (e.g., violence exposure, perceived stress, and duration of violence) provided unique, actionable information about particular outcomes, highlighting the importance of examining subscale scores where relevant. This aligns with other research supporting multidimensional models of adversity (Abravanel & Sinha, 2015; Myers et al., 2015) and underscores the bifactor model’s capacity to balance simplicity and complexity (Cooper & Nickodem, 2021). By summarizing the shared variance across dimensions while retaining the distinct contributions of specific adversities, the bifactor model helps guide decisions about whether to prioritize total scores, subscale scores, or both when interpreting and applying measures (Chen et al., 2012). Applying a bifactor model to childhood adversity advances the field by offering a framework to study both the cumulative impact of violence exposure and the unique effects of the emotional response to violence. This, in turn, supports the development of targeted interventions and policies tailored to address both broad and specific challenges associated with adversity (Cooper & Nickodem, 2021; Decrop et al., 2024).

### Childhood Adversity in High-Risk Communities

Childhood exposure to adversity, particularly in high-risk, under-resourced communities, is a critical area of research due to its profound and long-lasting impacts on mental health and developmental outcomes (Boyce, 2016; Nelson et al., 2020). Children in LMICs are estimated to be ten times more likely to be exposed to violence compared to their peers in HICs (Hillis et al., 2016). Exposure to violence, in particular, has been consistently linked to emotional distress responses, such as internalizing symptoms (e.g., anxiety and depression) and externalizing behaviors (e.g., aggression), which can significantly affect long-term mental health (Finkelhor et al., 2015; McLaughlin et al., 2014; Tsunga et al., 2024). Prior studies suggest that emotional distress responses may serve as a key mechanism through which violence exposure impacts long-term outcomes (Evans et al., 2013; Margolin & Gordis, 2004). For instance, heightened emotional reactivity may exacerbate the risks associated with violence exposure, leading to persistent behavioral or psychological difficulties (Evans et al., 2013). Variations in distress responses may explain why some children show resilience while others experience significant impairment following similar exposures (Masten, 2011). Understanding these relationships is particularly important in LMICs, where the burden of poor mental health among children and adolescents is disproportionately high (Lund et al., 2018; Patel et al., 2016). Children who face these problems early on in life are often faced with long-term negative consequences for themselves, their families, friends, and society at large (Cartwright et al., 2015). This current paper tried to give a detailed assessment of childhood adversity by combining two instruments that measure: (a) the child’s exposure to (community) violence and concurrently, (b) the child’s emotional distress response after a traumatic experience. Both instruments are part of the measurements done in the Drakenstein Child Health Study (DCHS), a multidisciplinary longitudinal birth cohort study investigating the determinants of child health in under-resourced communities outside Cape Town, South Africa (Zar et al., 2015). The DCHS is unique in that it is one of the first birth cohort studies globally to comprehensively investigate risk factors for child health (environmental, infectious, nutritional, genetic, maternal, and psychosocial; Stein et al., 2015).

### Rationale

The DCHS provided a unique opportunity to create a model of childhood adversity by exploring the combination of a child’s violence exposure and a child’s emotional distress response. Since adversity is often assumed in research (Kolar, 2011), this study aimed to specify adversity by creating and testing a new model of childhood adversity that builds on two established measures. We hypothesized that this would provide a specified assessment of childhood adversity for children in a high-risk context, by not only summing up a category of violence exposure but also tracking trauma-related behaviors in children who have been exposed to violence to account for interindividual differences in stress response (Nelson et al., 2020). There is a need for research into the shared effects of ACEs and how these effects are interrelated to adequately capture the psychological distress symptoms experienced by a child (Decrop et al., 2024). A multidimensional model that incorporates both shared and specific effects of adversities can advance our understanding of how adversity impacts children and inform the development of more precise and effective interventions (Abravanel & Sinha, 2015). Given the high covariance between exposure to violence and emotional distress, we hypothesized that a bifactor model would best fit the data (Evans et al., 2013). The following research questions were posed:

1. Can the underlying relationship between measured variables of violence exposure and emotional distress be identified to form a comprehensive measure of childhood adversity?
2. Can the structure that emerges from the combination of measures be confirmed with Confirmatory Factor Analysis to identify the relationship to overall childhood adversity?
a. Is a bifactor model the best fit for the data?

## Methods

### Study Site and Setting

This was a sub-study of a larger, ongoing birth cohort study (DCHS). For more in-depth information on this multidisciplinary birth cohort study, see Stein et al. (2015) and Zar et al. (2015). For more information on the psychosocial measures included in the cohort, see Donald et al. (2018). Pregnant women from two primary health care centers (TC Newman and Mbekweni clinics) were invited to join the DCHS. These health care centers are set in two communities with different cultural backgrounds. For example, participants from Mbekweni predominantly speak isiXhosa as their first language, whereas TC Newman participants mostly speak Afrikaans. Both abovementioned communities are stable, yet low socioeconomic status communities (Stein et al., 2015) and the cohort can be considered representative of other South African and LMICs peri-urban communities/settlements.

### Participants

**Figure 1.**
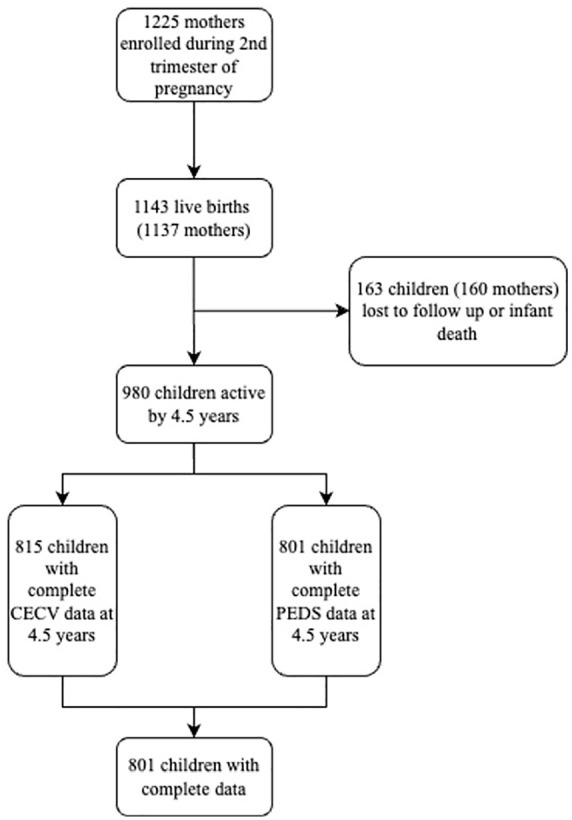
Flow chart of DCHS participation and missing data. *Note*. DCHS = Drakenstein Child Health Study.

Enrollment began in March 2012 and ended in March 2015; inclusion criteria were women at least 18 years of age, who were planning to stay in the area for at least 1 year, and planning to receive antenatal care at either of the two healthcare clinics. Mothers who consented were enrolled at 20 to 28 weeks gestation, and mother–child dyads have been followed longitudinally at several time points; currently, the oldest children are 12 years of age (June 2024). At enrollment, mothers provided informed written consent (guided by local staff and in the mother’s language of choice) and were further re-consented annually after childbirth. Mother–child dyads attended follow-up visits at the two clinics and Paarl Hospital (Stein et al., 2015). A total of 1,137 mother–child dyads were enrolled in the study, with 1,143 live births (4 sets of twins and one triplet). Due to attrition, the current sample in the DCHS is 980 (see Figure 1). Due to the late initiation of specifically the Child Exposure to Community Violence (CECV) and Pediatric Distress Scale (PEDS) data collection during the first study visit (age 3.5), our current sample size is smaller than the total DCHS cohort.

### Procedure

The administration of both the CECV and the PEDS was done at the 3.5- and 4.5-year timepoints, and the measures were completed by the mothers or an eligible caregiver (living with the child and/or looking after them for 3 or more days per week). The administration was in the home language of the participants and conducted by trained research assistants from the University of Cape Town. The measures were directly administered in Afrikaans for the Afrikaans-speaking participants and isiXhosa through the aid of interpreters for the isiXhosa-speaking participants, and English versions were available at all times. Both measures were translated using standard forward and back-translation processes and consensus meetings were held, including both communities to cross-check the translations to ensure the appropriate language and dialect were used. Study data were captured and managed using REDCap electronic data capture tools (Harris et al., 2009, 2019).

### Measures

#### Child Exposure to Community Violence

The CECV originally is a 39-item parent-report checklist, adapted from the widely used “Things I Have Seen and Heard Scale” that evaluates children’s exposure to violence (Amaya-Jackson, 1998). The questions contain information on the type and level of violence (domestic, school, community) that the child has witnessed or personally experienced (as victim or perpetrator). Higher scores indicate greater exposure to violence. The current version was adapted to fit the South African population more correctly, using examples of adversity that children in South African townships are exposed to, such as home robbery, assaults, stabbings, and gun shootings (Fincham et al., 2009). Four items from the original scale were deleted, and certain wording was modified for cultural relevance and clarity. Notably, items measuring perpetration of violence (e.g., “Has your child ever hurt someone else badly?”) and non-violence-related experiences (e.g., “Has your child ever witnessed drug deals in the community?” or “Has your child ever seen someone being arrested?”) were excluded (Kaminer, Hardy, et al., 2013). Example items on the CECV include “Has this child heard gunshots?” and “Has anyone at home ever hit this child so hard they were hurt?”. The answer possibilities were on a 4-point Likert scale (“*Never*”, “*Once*”, “*A few times*”, “*Many times*”). The total score for the CECV was calculated by summing the 35 items, with higher scores indicating a greater exposure to violence (range 0–105). Mean scores have ranged from 13.71 (*SD* = 10.75, range 0–116) in young children (aged 3–5 years) in a South African township (Cook et al., 2022) to a score of 32.92 (*SD* = 20.72, range 0–156) in South African adolescents (Fincham et al., 2009). Within the adapted version of the CECV, four subscales have been identified (Kaminer, Du Plessis, et al., 2013; Tsunga et al., 2023), namely, Witnessing Community Violence (10 items, α = .72), Community Victimization (8 items, α = .75), Witnessing Domestic Violence (6 items, α = .75), and Domestic Victimization (11 items, α = .79).

The CECV has shown good psychometric properties such as good internal consistency in previous South African studies, with α = .93 (Fincham et al., 2009) and α = .86 (Kaminer, Du Plessis, et al., 2013). In this study, the internal consistency of the CECV was assessed using McDonald’s omega (ω). The omega total was found to be .73, indicating acceptable reliability (*N* = 815). To our knowledge, the CECV has only been used in one other study (outside of the DCHS cohort) to measure the exposure to community violence of South African children under the age of 10 years (Cook et al., 2022). The internal consistency of the total score was acceptable with a Cronbach’s alpha of .72. The subscales (here described as three instead of four) demonstrated poor internal consistencies, with Cronbach’s alphas of .64, .36, and .17 for CECV factors 1, 2, and 3, respectively (Cook et al., 2022).

#### Pediatric Emotional Distress Scale

The PEDS is a brief screening tool for behavioral consequences related to the occurrence of traumatic events during childhood and is completed by a parent (Saylor et al., 1999). The PEDS asks about the child’s previous experience of a traumatic event and trauma-related experience of anxiety/withdrawal, fearfulness, and acting out/externalizing behaviors. There are three subscales: Anxious/Withdrawn (6 items, α = .74), Fearful (5 items, α = .72), and Acting Out (6 items, α = .78). The first part consisted of 17 items concerning general behavior (“Does your child have bad dreams?” or “Does your child have temper tantrums?”) with four answer possibilities (“*Almost Never*”, “*Sometimes*”, “*Often*”, & “*Very Often*”). The total score for the PEDS was calculated by summing all the items (range 17–68) of part 1. The second part consisted of four trauma-specific items, yet only item 18 (“Has the child had a major trauma”) has been included in the analysis for validity testing purposes. High scores on the 17 items (part 1) indicated a higher level of distress in the child. Mean scores on items 1 to 17 of the PEDS have varied greatly among research from as low as 21.9 (*SD* = 9.58, range 0–84; Saylor et al., 2003) and 25.7 (*SD* = 8.28, range 17–68; Coren et al., 2023) to as high as 52.3 (*SD* = 6.23, range 0–84; Bhushan & Sathya Kumar, 2009). Furthermore, factor structure analysis for the PEDS has shown different structures across studies (Cartwright et al., 2015; Coren et al., 2023; Saylor et al., 1999). Recent research proposed a cut-off score at the 90th percentile as a guideline for emotional and behavioral difficulties that warrant further evaluation (Coren et al., 2023). A good reliability was found for the PEDS scale with a McDonald’s omega (ω) of 0.87 (*N* = 803).

Research in a diverse sample of children 2 to 7 years of age showed that the PEDS is culturally sound and can be used in a variety of community contexts in the United States (Spilsbury et al., 2005). The total scale showed acceptable internal consistency, with a Cronbach’s alpha of .80, and the two subscales used here (Internalize subscale and Act Out subscale) showed acceptable internal consistency too, with Cronbach’s alpha of .80 and .82, respectively (Spilsbury et al., 2005) More recently, a study in the United States established nationally representative norms and percentiles for the PEDS (Coren et al., 2023). This study demonstrated a high internal consistency for the 17 core items (part 1), with a Cronbach’s alpha of .92, and acceptable internal consistency for the subscales (Coren et al., 2023)

### Ethics

Ethical approval was obtained from the Faculty of Health Sciences Research Ethics Committee, University of Cape Town (401/2009), and by the Western Cape Provincial Research Committee (2011RP45). When significant health issues were identified by study staff, mothers and children were referred to local healthcare services for further assessment and management. Given the sensitive content of both measures, a key obligation in the study was to flag instances of abuse, trauma, and mental health issues. An active referral system was in place for both mothers and children, supported by close relationships between study staff and provincial health staff. Furthermore, all women participating in the study, regardless of specific mental or physical health problems, were informed about social and support service providers available to them.

### Data Analysis

Data analysis was performed using R Statistical Software (version 4.1.2) and R Studio (version 2922.07.1) for Mac (R Core Team, 2022). The Exploratory factor analysis (EFA) was applied with a maximum likelihood estimator and an oblique rotation (Oblimin) to allow for correlated factors. A range of factor solutions was tested, starting with one factor and progressively increasing the number of factors. The selection of the optimal factor solution was guided by (a) Eigenvalues: Factors with eigenvalues greater than 1.0 were retained based on the Kaiser criterion, (b) Scree Plot: The scree plot was inspected visually to determine the “inflection point,” indicating where additional factors contributed diminishing explanatory variance, and (c) Theoretical Interpretability: Factors were evaluated for their conceptual relevance and alignment with theoretical constructs, and (d) Item Loadings: Items with loadings greater than 0.30 were retained (Field, 2009), and cross-loadings were monitored to ensure clarity in the factor structure. While both the CECV and PEDS have known subscales, prior studies have demonstrated variability in their factor structures across different populations and contexts (PEDS: Cartwright et al., 2015; Coren et al., 2023; Saylor et al., 1999, and CECV: Cook et al., 2022). We aimed to test whether the same variability was present in our dataset by first conducting EFAs for both measures separately. This allowed us to thoroughly examine item distribution and identify any patterns or inconsistencies specific to our sample. Using an exploratory-confirmatory approach ensured that our subsequent CFAs were based on empirical evidence from our data, rather than solely relying on predefined structures, which may not fully align with the unique characteristics of our study population. Confirmatory factor analysis (CFA) using Lavaan (Rosseel, 2012) was performed, making use of full information maximum likelihood (FIML) estimation when handling missing values. Also, a maximum likelihood estimator with robust standard errors (MLR) using a numerical integration algorithm was employed. The following goodness-of-fit indices were used to determine acceptability of the models/factor structures (cut-off scores are indicated in brackets): (a) chi-square (χ^2^) degrees of freedom (*df*), (b) the Tucker–Lewis index (TLI; 0.95), (c) the comparative fit index (CFI; 0.95), (d) root mean square error of approximation (RMSEA; 0.08), (e) standardized root mean square residual (SRMR; 0.08), and (f) the 90% confidence interval (CI; 0.08) of RMSEA and its significance (*p* < .06; Schreiber et al., 2006). In addition, the chi-square/*df* ratio ≤ 3 rule was also used (Kline, 2016). The factor structure identified through EFA was tested in CFA and compared against alternative models, as described below. The model with the best fit, as determined by the highest CFI/TLI and lowest RMSEA/SRMR, was selected:

Model of unitary dimension:

Model 1: One-factor CFA model (all items)

Models with subdomains:

Model 2: First-order CFA model (only subscales)
Model 3: Higher-order CFA model (subscales plus total score)
Model 4: Bifactor CFA model (subscales plus general factor)

The model with the best goodness-of-fit (i.e., highest CFI/TLI and lowest RMSEA/ SRMR) was then selected and factor scores were calculated for the new AERM model to calculate the overall adversity levels. Hierarchical models (and bifactor models in particular) tend to overfit the data, making comparisons of fit difficult (Chen et al., 2012; Markon, 2019). To account for potential overfitting of the data in the proposed models, total scores for each of the two individual measures were also included separately in the analysis to compare the best fit. Factor scores for the validity regressions were created using the standard Thurstone method, which constructs regression-based scores by applying the factor loadings as weights to the observed variables. To assess the internal validity of the resulting factor models, we tested associations between factor scores for the CECV, PEDS, and AERM total scores and responses to PEDS 18 (“Has the child had a major trauma”). Examples of traumatic events include taxi accidents, house/shack burning down, loss of a parent/family member, etc. This served as an external indicator of trauma-related behaviors, ensuring the constructs were relevant and interpretable.

To address missing data for the CECV and PEDS, imputation was performed using corresponding data from the 3.5-year timepoint to fill in missing values at the 4.5-year timepoint, maximizing the sample size and improving robustness for analyses. For CECV, the number of participants with complete data increased from 745 (pre-imputation) to 815 (post-imputation), with 37 participants remaining missing all items, due to missing data at both timepoints. Similarly, for PEDS, complete cases increased from 57 to 803 participants after imputation, with 35 participants missing all items and 11 participants showing partial missingness (1 item). This imputation process, resulting in an analytic sample size of 801 participants, substantially improved data completeness while maintaining transparency regarding unresolved missingness, ensuring the integrity of downstream analyses.

In under-resourced communities, such as those included in this study, violence exposure is pervasive, with approximately 75% to 80% of children exposed to multiple adverse experiences at a very young age (Tsunga et al., 2023, 2024). As a result, traditional cut-offs, such as those based on 4 or more adverse events commonly used in ACE studies, may not adequately capture the severity of exposure in this context (Evans et al., 2013). Instead, the 90th percentile cut-off was selected to identify children with the most severe cases of violence exposure and emotional response, representing those at the highest risk of negative outcomes. The 80% cut-off has been used as a slightly more moderate option. Finally, a binary yes/no indicator of exposure to violence for each CECV item was created (cecv_binary) by collapsing the answer options “*once*,”“*a few times*,” and “*many times*” to indicate “*yes*” to violence exposure and the rating “*never*” was retained as an indication of no exposure. This binary variable was used only for descriptive purposes and not in the main analyses.

## Results

### Descriptive Statistics

Table 1 shows sample characteristics for the 801 participants with completed PEDS and CEV data in the current sub-study. The vast majority of children (88%) came from households with a monthly income below R5,000 (272.57 U.S. Dollars), with 50% of the mothers reported being employed, and 37% completed secondary education.

**Table 1:** Sociodemographic and Child HIV Exposure Descriptive Statistics for the 4.5-year Timepoint.

Characteristic	*N* = 801^a^
Maternal age at birth	26.0 (22.0, 31.0)
Child sex	
Female	400 (50%)
Male	401 (50%)
Child HIV exposure	
HIV unexposed	632 (79%)
HIV exposed uninfected	167 (20.8%)
Infected	2 (0.2%)
Highest maternal educational achievement (2 levels)	
Lower than secondary	503 (63%)
At least secondary or higher	298 (37%)
Current parental employment	
Not working	402 (50%)
Working	399 (50%)
Current household income	
<R1,000/m	286 (36%)
R1,000–5,000/m	419 (52%)
>R5,000/m	96 (12%)

a Median (IQR); *n* (%).

### Child Emotional Distress Score

The mean score for the PEDS total score was 27.04 (*SD* = 7.11). In the original set of 801 observations at the 4.5-year aggregate time point, 7.74% of the participants had a score higher than 39 (the 90% cut-off score) on the PEDS. This percentage suggests a slightly lower rate of emotional disturbance compared to 5 to 6-year-old children in the U.S. (Coren et al., 2023). However, the 80% cut-off score (PEDS score > 31) was met by 23.35% of the children in the DCHS, which suggests a higher rate of emotional disturbance compared to their U.S. peers.

### Child Exposure to Violence

The mean score for the total score on the CECV was 3.46 (*SD* = 3.92). A 90% cut-off score on the CECV counts for a score higher than 8, which amounts to 10.61% of the 801 participants. This suggests that 10.61% of the children aged 4.5 years have been exposed to a multitude of violent events (community, domestic, or preschool). A score of 6 or higher on the CECV amounts to the top 20% at the 80th percentile, which includes 21.60% of the participants. A binary yes/no indicator of exposure to violence for each CECV item was created. This resulted in 74.66% of the participants having experienced a form of violence at least once in their life at 4.5 years of age, which corresponds with other findings of the CECV (Tsunga et al., 2023, 2024).

### Correlating the PEDS and CECV

**Figure 2.**
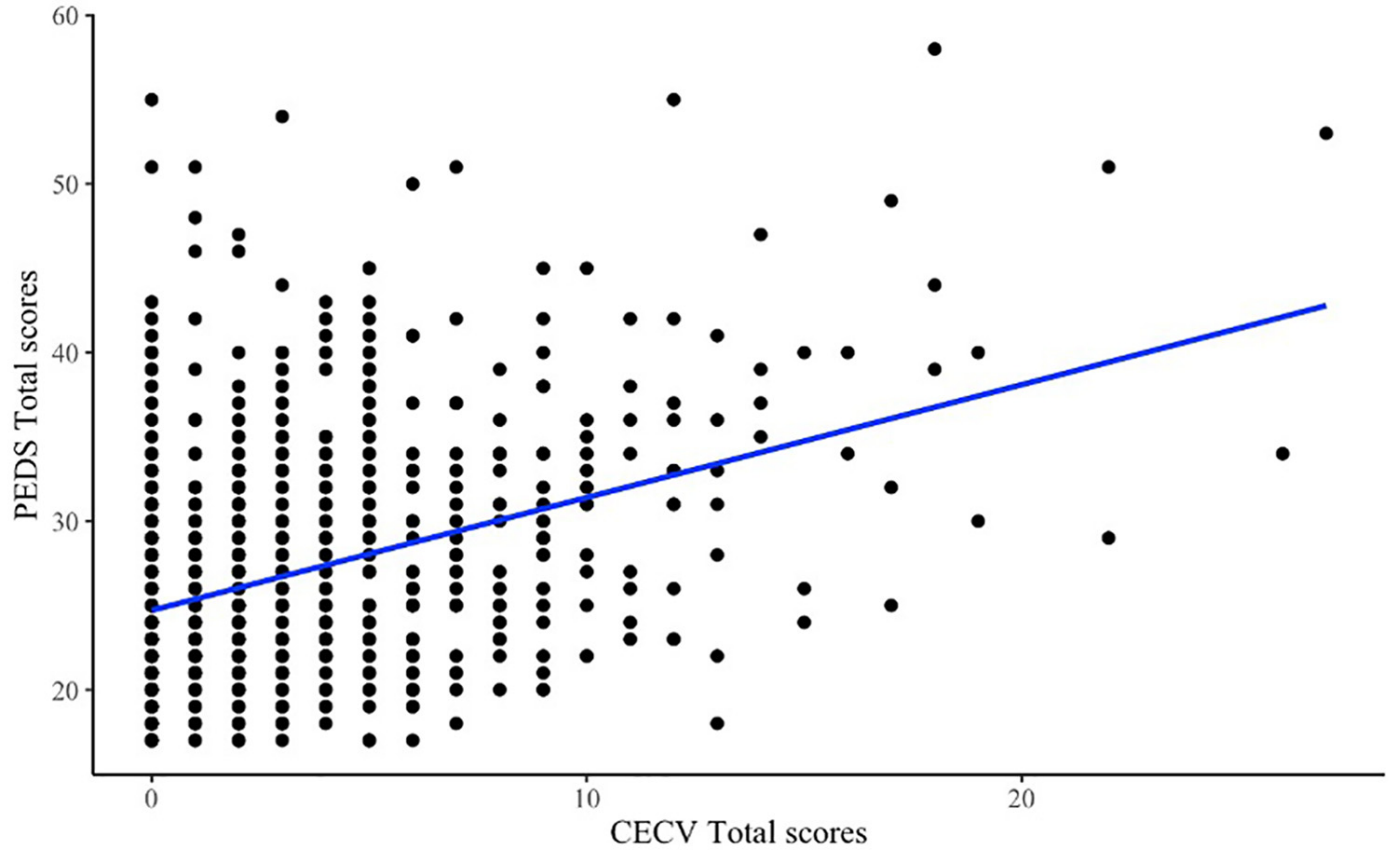
Scatter plot of the relationship between PEDS and CECV total scores. *Note*. Blue line is a least squares regression. CECV = Child Exposure to Community Violence; PEDS = Pediatric Emotional Distress Scale.

To look at the correlation between the total scores on the PEDS and the CECV, a Pearson correlation coefficient was computed. This revealed a small but positive correlation between the two variables, *r* (801) = 0.37, *p* < .001. The 95% confidence interval ranged from 0.31 to 0.43. The small correlation suggests that there is some relationship between the total scores on the PEDS and CECV, but more in-depth analysis was needed. As shown in Figure 2, one of the reasons for the weaker-than-expected correlation between the two adversity measures is that at the lowest levels of CECV scores, there is still a full range of PEDS scores, suggesting there is heterogeneity in the response to traumatic events.

A chi-squared test of independence was conducted to examine the relationship between the top 10% of children who experience emotional distress and the top 10% of children who experience exposure to violence. The analysis revealed a significant association between the two variables, χ^2^(*df* = 1, *N* = 801) = 26.19, *p* < .001. The effect size, as measured by Cramer’s *V*, was found to be 0.18 which is considered a small-to-medium effect size. Practically, this means that while children with high exposure to violence are more likely to experience significant emotional distress, the relationship is not deterministic, as not all children exposed to violence report high emotional distress. This aligns with our earlier finding of heterogeneity in the emotional responses to traumatic events, as seen in Figure 2, where a full range of PEDS scores was observed even at the lowest CECV levels.

Interestingly, a chi-squared test of independence between the top 10% of children with emotional distress and the cecv_binary highlighted a nonsignificant relationship between these two variables, χ^2^(*df* = 1, *N* = 801) = 2.51, *p* = .1133. It showed that 52 out of 62 participants with high levels of emotional distress reported an exposure to violence, yet 10 out of 62 participants with high levels of emotional distress, reported no exposure to violence at all. The participants with both exposure to violence and emotional distress account for 6.49% of the sample (52/801).

Another chi-squared test of independence was conducted to examine the relationship between the top 20% of children who experience emotional distress and the top 20% of children who experience exposure to violence. This analysis also revealed a significant association between the two variables, χ^2^(*df* = 1, *N* = 801) = 30.28, *p* < .001. The effect size, as measured by Cramer’s V, was found to be 0.19 and is considered a small-to-medium effect size.

### Research Question 1

#### Exploratory Factor Analysis

Both the CECV (*N* = 815) and PEDS (*N* = 803) data were subjected to an EFA with oblique rotation (oblimin). First, the CECV data was entered and the maximum likelihood factor analysis with a cut-off point of 0.30 and the Kaiser’s criterion of eigenvalues greater than 1 (Field, 2009; Werner et al., 2014) yielded a one-factor solution as the best fit for the data, with 17% of the variance explained. This initial analysis was conducted using all CECV items, but it became clear that several invariant items did not contribute meaningfully to the factor structure due to their lack of variability. To get to the one-factor solution, 23 items for the CECV were deleted due to invariance as they showed no variability in responses, with a standard deviation of less than 0.1, leaving 10 items in this first analysis (see Appendix 1). These items were likely not endorsed due to the young age of participants and the specific contextual factors of the sample, such as reduced likelihood of exposure to certain adversities. The remaining 10 items primarily corresponded to the “Witnessing Community Violence” subscale, as identified in previous studies on the CECV(Cook et al., 2022; Kaminer, Du Plessis, et al., 2013).

All 17 items of the PEDS were entered and the maximum likelihood factor analysis with a cut-off point of 0.30 and the Kaiser’s criterion of eigenvalues greater than 1 (Field, 2009; Werner et al., 2014) yielded a one-factor solution as best fit for the data, with 26% of the variance explained. Theoretically, a three-factor structure is suggested (Saylor et al., 1999; Spilsbury et al., 2005). This three-factor structure explained 36% of the variance in the current dataset. Interestingly, when examining the item distribution within the three factors, their structure was different than found in previous studies with the PEDS (Saylor et al., 1999; Spilsbury et al., 2005). See Table 2 for a comparison of the original PEDS factor structure and the current factor structure and Appendix 2 for a more detailed overview of the items per factor structure.

**Table 2:** Items for the PEDS in the Original Factor Structure and the Current Factor Structure. Items in bold Match Across Factor Structures.

Original Factor Structure			Current Factor Structure		
Factor 1 Acting Out	Factor 2 Fearful	Factor 3 Anxious/Withdrawn	Factor 1 Emotional Response	Factor 2 Fearful	Factor 3 Acting Out
1	3	6	1	**5**	**12**
2	4	7	2	**6**	**13**
11	**5**	8	4	7	14
**12**	**6**	9	**8**	9	**17**
**13**	**10**	14		**10**	
**17**		15		16	
		16			

*Note*. PEDS = Pediatric Emotional Distress Scale.

Finally, the remaining items for the CECV (10) and PEDS were merged to form the AERM with 27 items remaining (*N* = 801). The Kaiser–Meyer–Olkin (KMO) measure verified the sampling adequacy of our merged dataset for analysis, KMO = 0.86 (meritorious). Bartlett’s test of sphericity χ^2^(276) = 4,703.22, *p* < .001 indicated that the correlation structure was adequate for factor analyses. The maximum likelihood factor analysis with a cut-off point of 0.30 and the Kaiser’s criterion of eigenvalues greater than 1 (Field, 2009; Werner et al., 2014) yielded a two-factor solution as the best fit for the data, accounting for 27% of the variance. However, the screen plot indicated a four-factor solution, accounting for 34% of the variance, in line with the three factors for the PEDS and the one remaining CECV factor. In the final analysis with the merged data, 23 items remained (see Appendix 3 for an overview of the EFA for the merged data). While a two-factor model initially seemed intuitive given that the combined EFA included items from two separate scales (CECV and PEDS), the four-factor model was ultimately chosen because it provided a better fit to the data and explained more variance. Importantly, the four-factor model ensured that the unique contributions of CECV items were not overshadowed by the larger number of items from the PEDS, thereby preserving the specificity of each measure within the combined structure.

### Research Question 2

#### Confirmatory Factor Analysis

**Figure 3.**
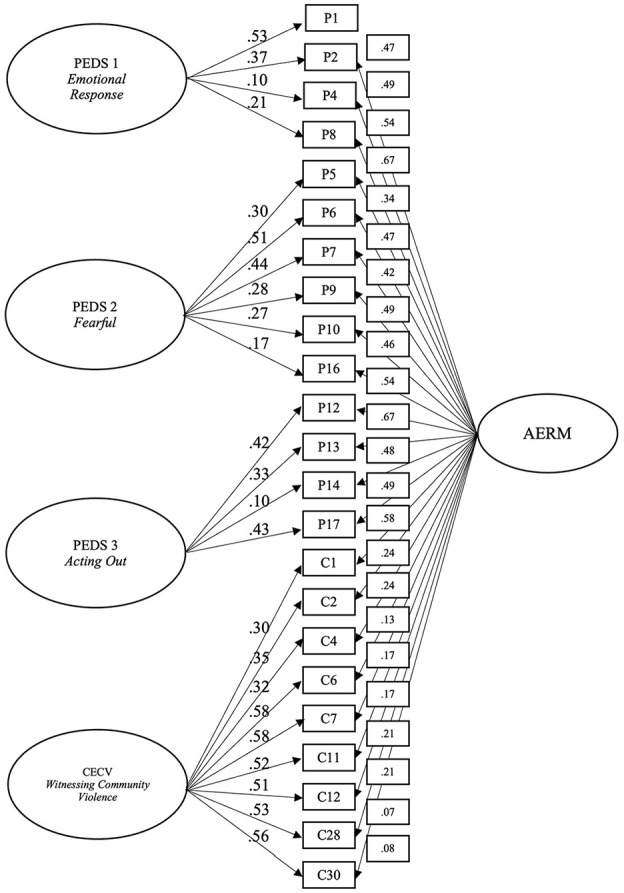
Bifactor model of the Adversity Exposure-Response Model with factor loadings per item.

Four models were examined to assess their goodness-of-fit, (a) one-factor CFA model, (b) first-order CFA model, (c) higher-order CFA model, and (d) bifactor CFA model. The results (see Table 3) showed that a bifactor model fits the data better than the other three models, suggesting that within the bifactor model, there is a general factor (supposedly adversity) that is separable from the specific factors (Emotional Response, Fearful, Acting Out [PEDS], and Witnessing Community Violence [CECV]). The fit indices are acceptable for the RMSEA (<0.08), and SRMR (<0.08), but inadequate for the CFI and TLI (<0.95). Figure 3 shows the final fit for the bifactor model. Of note, some of the items in the bifactor model loaded weakly/moderately onto either the general factor or on their respective subscale; however, literature has not established cut-offs for acceptable factor loadings in bifactor models (Decrop et al., 2024; Reise et al., 2010). All but two items (cecv_28; “*Has this child known someone that was killed by another person?”* and cecv_30 “*Has this child seen someone being killed by another person at home?*”) loaded significantly onto the general factor (see Appendix 4 for full description of the bifactor model).

**Table 3:** Fit of the Four-Factor Models to the Adversity Exposure-Response Model.

Model	χ^2^	*df*	CFI	TLI	RMSEA	SRMR
(i) One-factor model	1,251.10	230	0.635	0.599	0.090	0.090
(ii) First-order factor model	576.702	224	0.888	0.873	0.051	0.048
(iii) Higher-order factor model	577.593	226	0.888	0.875	0.050	0.049
(iv) Bifactor model	545.315	207	0.903	0.882	0.049	0.042

*Note*. CFI = comparative fit index; TLI = Tucker–Lewis index; RMSEA = root mean square error of approximation; SRMR = standardized root mean squared residual.

A score for the AERM was formed by summing the factor scores for the 3 PEDS factors as well as the CECV factor score. This variable ( $\bar{x}$ = 5.15, *SD* = 2.58) showed strong correlations with the peds_total (*r* = .46, *p* < .001) and cecv_total (*r* = .60, *p* < .001) as well as with the peds subscales (*r* = .23, *r* = .36, *r* = .22, and *p* < .001 for all), and the CECV subscale (*r* = .70, *p* < .001). This is expected as it was aggregated from PEDS and CECV subscales scores.

#### Internal Validation of the Adversity Exposure-Response Model

To evaluate the relationship between item PEDS 18 (“Has the child had a major trauma?”) and the exposure to violence, a chi-squared test of independence was conducted. The analysis indicated a significant relationship between peds_18 and CECV 90% percentile, χ^2^(*df* = 1, *N* = 801) = 26.61, *p* < .001. The effect size, as measured by Cramer’s *V*, was found to be 0.18 and is considered a small-to-medium effect size. These findings provide support for the validity of the AERM by demonstrating consistent associations between emotional distress and increasing levels of violence exposure.

Children whose mothers reported “*yes*” on the trauma/stress item PEDS 18 (*N* = 72) were 2.67 times more likely to score above the 90% cut-off score on the PEDS compared to those children (*N* = 729) that did not report a traumatic event, 18.1% versus 6.72%. This showcases strong internal validity, where children who have experienced a traumatic event had higher scores on the PEDS than those without maternal-reported trauma exposure.

To conclude, three logistic regression models were fitted to examine the relationship between Peds_18 and various predictors, including (a) the factor scores for the three PEDS subscales (PEDS1-3) and the CECV factor, (b) the total scores of the CECV and PEDS scales, and (c) the AERM score (see Tables 4 and 5 for results). Results revealed that the total score on the CECV was a significant positive predictor (β = .15, *p* < .001), as was the total PEDS score, although its effect was negative (β = −.01, *p* = .040). When examining the factor scores individually, PEDS factors 2 and 3 (Fearful and Acting Out, respectively) were significant predictors (β = .39, *p* = .022; β = .49, *p* = .010), alongside the CECV factor (β = .38, *p* < .001). Notably, the newly developed AERM score was also a statistically significant predictor of trauma exposure (β = .34, *p* < .001). These findings suggest that while individual scales and factors contribute unique information, the AERM effectively synthesizes this data into a robust metric for predicting trauma exposure.

**Table 4:** Correlations Between the Four Subdomains, the Bottom Diagonal Shows Correlations, and the Top Diagonal *p*-values.

Variables	PEDS 1	PEDS 2	PEDS 3	CECV
PEDS 1 *Emotional Response*	1.00	<.001	<.001	<.05
PEDS 2 *Fearful*	−.24	1.00	<.001	ns
PEDS 3 *Acting Out*	−.19	−.26	1.00	ns
CECV *Witnessing Community Violence*	.08	.04	.04	1.00

*Note*. CECV = Child Exposure to Community Violence; PEDS = Pediatric Emotional Distress Scale.

**Table 5:** Factor Scores and Total Scores on the CECV and PEDS, and the Adversity Exposure-Response Model, as Potential Predictors of a Direct Measurement of Trauma Exposure.

Item	Coefficient	Std. Error	*Z*-value	*p*-value	AIC
(Intercept)	−9.45	1.06	−8.88	<.001*	437.63
total_cecv	0.15	0.06	0.90	<.001*	
total_peds	−0.01	0.02	−0.241	.040*	
(Intercept)	−2.43	0.13	−18.16	<.001*	468.84
PEDS1	−0.17	0.01	−1.43	.455*	
PEDS2	0.39	0.21	1.42	.022*	
PEDS3	0.49	0.20	1.77	.010*	
CECV	0.38	0.15	0.14	<.001*	
(Intercept)	−2.39	0.13	−18.48	<.001*	470.76
AERM	0.34	0.08	4.18	<.001*	

*Note*. AERM = Adversity Exposure-Response Model; CECV = Child Exposure to Community Violence; PEDS = Pediatric Emotional Distress Scale.*p < .05.

## Discussion

This study aimed to form a comprehensive measure of childhood adversity, by combining the child’s exposure to violence and emotional distress response. The main findings were (a) a bifactor model demonstrated a novel approach to measuring childhood adversity in a high-risk South African birth cohort study through the integration of two existing measures, (b) this bifactor model identified a general adversity factor that was able to predict direct trauma exposure in children, and (c) four subscales were identified, representing distinct dimensions of a violence exposure domain and emotional distress response domains.

The bifactor approach was useful in transcending the assumption that all adverse events result in uniformly negative outcomes, moving beyond the traditional ACEs framework. With results showing that almost 75% of the children were exposed to community violence, the percentage of children with reported signs of elevated distress was 8%, with 6.5% of the sample reporting high scores on both constructs. This heterogeneity in emotional responses to the experience of trauma underscored the importance of combining the two measures, as it highlighted the variability in responses to similar levels of violence exposure. The AERM displayed exposure to community violence and levels of emotional distress response; hence, it can account for interindividual differences in response. By explicitly linking exposure to violence with its emotional impact, the AERM provides a valuable contribution to the literature, illustrating how children process ongoing violence and reinforcing the need for assessments that account for both risk and resilience.

Consistent with prior studies (e.g., Abravanel & Sinha, 2015; Cooper & Nickodem, 2021; Myers et al., 2015), our findings underscore the multifaceted nature of ACEs. However, by using a bifactor model, we were able to identify a general adversity factor that provides a more comprehensive assessment of risk compared to using subscales or single measures alone. An important validity check for the use of the adversity composite is the fact that it is a significant predictor of direct trauma exposure in children. The AERM reflects the cumulative burden of violence exposure and emotional distress response, aligning conceptually with the goal of measuring overall trauma exposure. This links back to the importance of a bifactor model, that in this case, hypothesizes that there is one general factor that accounts for the commonalities across all childhood adversity variables—much like the “*g*” factor of general intelligence. The fact that all but two items loaded significantly onto the general factor, indicated that the CECV and PEDS have much in common.

While a two-factor model (CECV and PEDS) initially appeared logical, a four-factor model was ultimately selected for its superior fit and explanatory power. Although the bifactor model’s fit indices were not exceptionally strong, they were significant and generally above the cut-off thresholds, making this exploratory approach justifiable in the context of developing a novel framework. Importantly, the choice for a four-factor model ensured the CECV items retained their unique contributions, rather than being overshadowed by the PEDS’ larger item count, thereby preserving the specificity of both measures. Furthermore, as the PEDS is known to have a multifactor structure, the four-factor model captured this complexity, enabling a nuanced representation of distinct dimensions of violence exposure and emotional distress. This methodological choice strengthens the model’s capacity to account for the multifaceted nature of childhood adversity.

When looking at the individual measures, the PEDS mean score ( $\bar{x}$ = 27.04, *SD* = 7.11) was slightly higher than research into a national sample in the U.S. ( $\bar{x}$ = 25.7, *SD* = 8.28; Coren et al., 2023). However, when comparing the age-group-specific data (4.5y in the current dataset versus 5–6 years in the U.S.) the data was more or less the same ( $\bar{x}$ = 27.04 in SA vs. $\bar{x}$ = 27.5 in the U.S.). Interestingly, in our high-risk cohort, the percentage of children that scored above the 90% cut-off score was lower, which would indicate that fewer children are showing signs of emotional disturbance compared to their peers in the U.S. Younger children tend to score higher on the PEDS (Coren et al., 2023), even though they might not have been exposed to more trauma or stress, indicating that in young children, behavior such as bed-wetting, clinging to adults, or having temper tantrums is regarded normal and occurs more frequently.

Furthermore, the average CECV was low ( $\bar{x}$ = 3.46, *SD* = 3.92; range 0–105) indicating that the frequency of the exposure was low. This seems low compared to other research; for instance, in young children in South Africa coming from the same age (3–5 years old) and high-risk background ( $\bar{x}$ = 13.71, *SD* = 10.75, range 0-116; Cook et al., 2022). Yet, 75% of our young participants were exposed to at least one incidence of community violence in their young lives, which corresponds to other research in the same context (Cook et al., 2022; Tsunga et al., 2023).

The fact that children in the DCHS cohort were showing fewer signs of emotional disturbance despite reporting high rates of violence exposure was unexpected, given the high-risk areas these children grow up in and in general, South African children are exposed to disproportional violence levels (Meinck et al., 2016). Perhaps, the mothers in this cohort have (un)intentionally tried to minimize the reporting of their child’s exposure to adversity (i.e., due to social desirability, since assessment was done via caregiver recall and report, and administered via in-person interviewers)—some research showed that mothers tend to under-report on the frequency and severity of these adverse events (Fisher et al., 2011), especially when some of the questions are relating to their own parenting practices or are related to domestic violence. The relatively low score for child violence exposure and emotional distress could also stem from a prolonged exposure to trauma—linking back to the theories of desensitization or habituation, with prolonged exposure to frequent and severe adversity leading children to normalize such experiences, reducing the perception of emotional disturbance in response to these events (Kaminer & Eagle, 2010). This could explain the lower levels of reported emotional disturbance despite 75% of these 4.5-year-olds being reported having been exposed to a traumatic event in their young life at least once already. An alternative explanation can be that these results show posttraumatic growth, even at this young age. This could be in line with the vulnerability paradox in posttraumatic stress disorder (PTSD; Dückers et al., 2016), which states that greater country vulnerability is associated with decreased risk of PTSD for citizens, stating an important role for cultural and psychological factors beyond mere resource availability. Nonetheless, it is concerning that the vast majority of young children were exposed to traumatic events early on in their formative years, and combined with heightened emotional distress, the potential association with PTSD or other behavioral problems seems obvious (Kaminer, Du Plessis, et al., 2013). However, in the South African context, we also must not forget the extraordinary strengths displayed by so many children and their ability to adapt positively to ever-challenging circumstances. Future research should be directed toward uncovering protective pathways and factors that bolster resilience despite these adversities (Masten & Cicchetti, 2010). Within the context of the DCHS, this adversity m.odel at the 4.5-year time point can be a critical element to understand the link between adversity and the subsequent child health outcomes.

### Limitations

The results of this study must be considered within the context of some limitations. As mentioned earlier, since all information was gathered via caregiver reports, with a translator and/or research assistant present at the time of assessment, it is not clear whether social desirability may have influenced the likelihood of accurate reporting. Only the items pertaining to the child’s witnessing of community violence were included in the analysis, yet the full CECV looks at different factors of violence beyond witnessing community violence (victimization of community/domestic violence and witnessing domestic violence). However, examination of these various types of violence exposures was unattainable due to the very low endorsement rates. Violence in the domestic context seems to be underreported since an earlier report shows that 27% of the women reported exposure to intimate partner violence within the DCHS cohort (Barnett et al., 2022). This could be attributed to social desirability bias again or perhaps mothers underestimated the fact that the young children were witness to this domestic abuse.

Using the PEDS in a high-risk/low-income setting in an LMIC revealed notable differences in item distribution across its subscales (see Table 1). While the PEDS was developed to evaluate children directly after a traumatic event, this was not the case in the DCHS with a scheduled assessment regardless of the child’s exposure to trauma, which could have led to different answer patterns than in the context of acute trauma. A methodological limitation was that there is no information on the exact timing of the exposure to violence and emotional distress response, making a longitudinal analysis impractical which led us to opt for creating an adversity model.

Future research should aim to uncover why certain children showed high levels of emotional distress response without exposure to violence, perhaps child temperament is of influence here. Furthermore, the correlation between the two measures was only of moderate effect, leading to a significant but not extraordinarily strong bifactor model. The fact that most items were taken from the PEDS, could have impacted the adversity composite. Finally, gender and cultural differences are an important future direction of research, particularly given the potential influence of societal norms on emotional expression and experiences of adversity. However, given the exploratory nature of this paper and the lack of instruments that accurately measure the multifaceted construct of adversity (Oh et al., 2018), any novel contribution should be welcomed.

## Conclusion

This study demonstrates the successful development of a bifactor model for adversity, with a general adversity factor and four subscales each contributing uniquely to understanding childhood adversity. Instead of solely tracking the frequency or severity of adverse events, the AERM combined exposure to community violence and emotional distress response. Given the potential long-term consequences of emotional distress, the AERM was able to provide valuable insight into how children process emotional distress and respond to violence. Since research into childhood adversity often leaves out the emotional distress response, the AERM is providing a valuable contribution to the literature. Practical implications include the potential for early identification of at-risk children using a comprehensive adversity framework, which can guide targeted interventions in high-risk settings. Future research can investigate the applicability of this model to other cohorts and explore its association with outcomes such as resilience or externalizing/internalizing behavioral problems, to further study its utility.

## Appendix

**Appendix 1.** Exploratory Factor Analysis of the CECV.

Items	Factor 1 (Witnessing Community Violence)	Communality
cecv_1_54mon	**0.40**	1
cecv_2_54mon	**0.46**	1
cecv_3_54mon	0.29	1
cecv_4_54mon	**0.36**	1
cecv_5_54mon	0.05	1
cecv_6_54mon	**0.57**	1
cecv_7_54mon	**0.52**	1
cecv_8_54mon	0.28	1
cecv_9_54mon	0.05	1
cecv_10_54mon	0.07	1
cecv_11_54mon	**0.58**	1
cecv_12_54mon	**0.53**	1
cecv_13_54mon	0.03	1
cecv_14_54mon	0.02	1
cecv_15_54mon	0.15	1
cecv_16_54mon	−0.02	1
cecv_17_54mon	0.11	1
cecv_18_54mon	0.09	1
cecv_19_54mon	0.05	1
cecv_20_54mon	0.12	1
cecv_21_54mon	0.03	1
cecv_22_54mon	/	1
cecv_23_54mon	/	1
cecv_24_54mon	/	1
cecv_25_54mon	0.10	1
cecv_26_54mon	/	1
cecv_27_54mon	0.08	1
cecv_28_54mon	**0.48**	1
cecv_29_54mon	0.17	1
cecv_30_54mon	**0.48**	1
cecv_31_54mon	0.28	1
cecv_32_54mon	0.26	1
cecv_33_54mon	0.21	1
cecv_34_54mon	0.13	1
cecv_35_54mon	**0.34**	1

*Notes*. Extraction method = maximum likelihood, rotation method = oblimin with Kaiser normalization. Loadings larger than 0.30 are in bold. CECV = Child Exposure to Community Violence.

**Appendix 2.** Exploratory Factor Analysis of the PEDS.

Items	Factor 2 (Fearful)	Factor 1 (Acting Out)	Factor 3 (Emotional Response)	Communality
peds_q1_54mon	0.01	0.10	**0.53**	1.08
peds_q2_54mon	−0.02	−0.02	**0.74**	1.00
peds_q3_54mon^a^	0.17	0.03	0.29	1.64
peds_q4_54mon	0.25	0.07	**0.36**	1.85
peds_q5_54mon	**0.39**	0.11	−0.03	1.18
peds_q6_54mon	**0.69**	−0.06	0.02	1.01
peds_q7_54mon	**0.62**	−0/05	0.00	1.01
peds_q8_54mon	0.24	0.22	**0.33**	**2.66**
peds_q9_54mon	**0.51**	0.18	−0.10	1.33
peds_q10_54mon	0.47	0.04	0.09	1.09
peds_q11_54mon^a^	−0.08	0.28	0.24	**2.13**
peds_q12_54mon	0.01	**0.73**	0.07	1.02
peds_q13_54mon	−0.02	**0.55**	0.08	1.04
peds_q14_54mon	0.19	**0.31**	0.08	1.81
peds_q15_54mon^a^	0.18	0.09	−0.03	1.50
peds_q16_54mon	**0.38**	0.18	0.10	1.57
peds_q17_54mon	**0.38**	0.18	0.10	1.57

*Notes*. Extraction method = maximum likelihood, rotation method = oblimin with Kaiser normalization. Loadings larger than 0.30 are in bold. PEDS = Pediatric Emotional Distress Scale.

a Are the items with factor loadings <0.30 across all proposed factors.

**Appendix 3.** Exploratory Factor Analysis of the AERM.

Items	Factor 2 (Witnessing Community Violence)	Factor 1 (Acting Out)	Factor 3 (Fearful)	Factor 4 (Emotional Response)	Communality
cecv_1_54mon	**0.32**	−0.03	0.00	0.24	1.88
cecv_2_54mon	**0.37**	0.08	−0.05	0.14	1.41
cecv_4_54mon	**0.32**	−0.02	0.06	0.03	1.11
cecv_6_54mon	**0.59**	−0.02	−0.04	0.10	1.07
cecv_7_54mon	**0.58**	0.05	0.06	−0.09	1.09
cecv_11_54mon	**0.56**	0.03	−0.11	0.18	1.30
cecv_12_54mon	**0.54**	0.03	0.00	0.05	1.02
cecv_28_54mon	**0.55**	0.04	0.00	−0.13	1.13
cecv_30_54mon	**0.57**	−0.07	0.16	−0.14	1.31
cecv_35_54mon^a^	0.24	0.09	−0.09	0.22	**2.54**
peds_q1_54mon	0.02	0.15	0.07	**0.45**	1.29
peds_q2_54mon	0.02	0.04	0.05	**0.66**	1.02
peds_q3_54mon^a^	−0.04	0.06	0.23	0.22	**2.21**
peds_q4_54mon	−0.03	0.08	0.28	**0.36**	2.04
peds_q5_54mon	0.12	0.12	**0.37**	−0.07	1.53
peds_q6_54mon	0.01	−0.04	**0.64**	0.07	1.03
peds_q7_54mon	0.11	−0.05	**0.59**	0.02	1.08
peds_q8_54mon	0.03	0.24	0.25	**0.33**	**2.80**
peds_q9_54mon	−0.04	0.18	**0.50**	−0.08	1.34
peds_q10_54mon	−0.07	0.06	**0.50**	0.09	1.13
peds_q11_54mon^a^	0.04	0.29	−0.06	0.18	1.72
peds_q12_54mon	−0.01	**0.76**	0.00	0.05	1.01
peds_q13_54mon	−0.01	**0.56**	0.00	0.04	1.01
peds_q14_54mon	0.01	**0.33**	0.18	0.07	1.67
peds_q15_54mon^a^	−0.04	0.10	0.21	−0.07	1.80
peds_q16_54mon	−0.01	0.18	**0.38**	0.11	1.64
peds_q17_54mon	0.04	**0.74**	0.01	−0.07	1.02

*Notes*. Extraction method = maximum likelihood, rotation method = oblimin with Kaiser normalization. Loadings larger than 0.30 are in bold. AERM = Adversity Exposure-Response model; CECV = Child Exposure to Community Violence; PEDS = Pediatric Emotional Distress Scale.

a Items with factor loadings <0.30.

**Appendix 4.** Confirmatory Factor Analysis of the AERM.

Item	Estimate	Std. Error	*p*(>|*z*|)	Std. All
Total =~				
peds_q1_54mon	0.412	0.039	.000	0.470
peds_q2_54mon	0.491	0.097	.000	0.491
peds_q4_54mon	0.520	0.096	.000	0.539
peds_q5_54mon	0.180	0.035	.000	0.344
peds_q6_54mon	0.247	0.032	.000	0.468
peds_q7_54mon	0.191	0.028	.000	0.417
peds_q8_54mon	0.519	0.043	.000	0.671
peds_q9_54mon	0.249	0.037	.000	0.476
peds_q10_54mon	0.353	0.043	.000	0.460
peds_q12_54mon	0.564	0.043	.000	0.671
peds_q13_54mon	0.411	0.039	.000	0.481
peds_q14_54mon	0.339	0.047	.000	0.492
peds_q16_54mon	0.326	0.038	.000	0.536
peds_q17_54mon	0.399	0.042	.000	0.579
cecv_1_54mon	0.273	0.058	.000	0.241
cecv_2_54mon	0.247	0.047	.000	0.237
cecv_4_54mon	0.047	0.022	.030	0.129
cecv_6_54mon	0.057	0.018	.001	0.171
cecv_7_54mon	0.037	0.013	.003	0.166
cecv_11_54mon	0.118	0.031	.000	0.212
cecv_12_54mon	0.083	0.023	.000	0.205
cecv_28_54mon	0.015	0.010	.161	0.069
cecv_30_54mon	0.012	0.008	.127	0.083
PEDS1 =~				
peds_q1_54mon	0.459	0.483	.342	0.525
peds_q2_54mon	0.371	0.451	.411	0.371
peds_q4_54mon	0.097	0.382	.801	0.100
peds_q8_54mon	0.166	0.084	.048	0.214
PEDS2 =~				
peds_q5_54mon	0.156	0.045	.001	0.298
peds_q6_54mon	0.270	0.051	.000	0.511
peds_q7_54mon	0.201	0.039	.000	0.438
peds_q9_54mon	0.145	0.051	.004	0.277
peds_q10_54mon	0.207	0.069	.003	0.270
peds_q16_54mon	0.104	0.066	.117	0.171
PEDS3 =~				
peds_q12_54mon	0.353	0.099	.000	0.420
peds_q13_54mon	0.285	0.060	.000	0.334
peds_q14_54mon	0.067	0.094	.479	0.096
peds_q17_54mon	0.299	0.060	.000	0.433
CECV =~				
cecv_1_54mon	0.336	0.066	.000	0.296
cecv_2_54mon	0.364	0.053	.000	0.350
cecv_4_54mon	0.116	0.036	.001	0.318
cecv_6_54mon	0.194	0.041	.000	0.579
cecv_7_54mon	0.128	0.042	.002	0.576
cecv_11_54mon	0.290	0.045	.000	0.521
cecv_12_54mon	0.206	0.051	.000	0.511
cecv_28_54mon	0.112	0.028	.000	0.527
cecv_30_54mon	0.078	0.034	.024	0.559

*Note*. AERM = Adversity Exposure-Response model; CECV = Child Exposure to Community Violence; PEDS = Pediatric Emotional Distress Scale.
